# Is obesity related to enhanced neural reactivity to visual food cues? A review and meta-analysis

**DOI:** 10.1093/scan/nsaa113

**Published:** 2020-08-12

**Authors:** Filip Morys, Isabel García-García, Alain Dagher

**Affiliations:** Department of Neurology and Neurosurgery, Montreal Neurological Institute, McGill University, Montreal, Quebec, Canada; Department of Neurology and Neurosurgery, Montreal Neurological Institute, McGill University, Montreal, Quebec, Canada; Department of Clinical Psychology and Psychobiology, University of Barcelona, Barcelona, Spain; Department of Neurology and Neurosurgery, Montreal Neurological Institute, McGill University, Montreal, Quebec, Canada

**Keywords:** obesity, cue reactivity, food cues, meta-analysis, fMRI

## Abstract

Theoretical work suggests that obesity is related to enhanced incentive salience of food cues. However, evidence from both behavioral and neuroimaging studies on the topic is mixed. In this work, we review the literature on cue reactivity in obesity and perform a preregistered meta-analysis of studies investigating effects of obesity on brain responses to passive food pictures viewing. Further, we examine whether age influences brain responses to food cues in obesity. In the meta-analysis, we included 13 studies of children and adults that investigated group differences (obese *vs* lean) in responses to food *vs* non-food pictures viewing. While we found no significant differences in the overall meta-analysis, we show that age significantly influences brain response differences to food cues in the left insula and the left fusiform gyrus. In the left insula, obese *vs* lean brain differences in response to food cues decreased with age, while in the left fusiform gyrus the pattern was opposite. Our results suggest that there is little evidence for obesity-related differences in responses to food cues and that such differences might be mediated by additional factors that are often not considered.

## Introduction

Individuals vary in their susceptibility to obesity. An oft-proposed explanation is that an environment abundant in appetizing food cues triggers different levels of hunger and eating in different people. According to this explanation, individuals more susceptible to omnipresent food cues might overconsume palatable foods and have difficulty to restrict their caloric intake, which may lead to obesity (Polivy *et al.*, 2008). However, there are few systematic reviews of cue-reactivity in obesity that unequivocally support the model.

The underlying theory is that foods and food-related cues can come to act as conditioned stimuli that predict the rewarding effects of ingestion. Food constituents, such as calories (Sclafani *et al.*, 2011), are unconditioned stimuli that elicit unconditioned metabolic responses (Jansen, 1998; Hill, 2007). When reliably paired with caloric intake, food cues such as the sight or smell of food might become conditioned cues that ultimately invoke conditioned responses such as hunger, food craving, salivation or brain activity (Jansen, 1998; Hill, 2007; van den Akker *et al.*, 2014). Several researchers have proposed that reactivity to food cues forms part of a trait that combines enhanced appetitive drive and reduced inhibitory control, which renders some individuals vulnerable to uncontrolled eating in an obesogenic environment (Vainik *et al.*, 2019).

Some studies have related reactivity to food cues to high body mass index (BMI) and obesity (Stice *et al.*, 2009 2010; Demos *et al.*, 2012; van den Akker *et al.*, 2014; Pursey *et al.*, 2014). However, the literature contains a number of conflicting findings on the topic (see Boswell and Kober, 2016). This is largely due to the diversity of paradigms and outcome measures used. Classically, one of the main outcome measures in food cue reactivity paradigms is self-reported cue-induced food craving, which is a strong and conscious desire to eat (Jansen, 1998; Jansen *et al.*, 2011; Boswell and Kober, 2016). It is different from trait craving, which is seen as a desire to eat arising independently of any external cues (Cepeda-Benito *et al.*, 2000; Boswell and Kober, 2016). Both types of cravings can be measured with standardized questionnaires, such as the State and Trait Food-Cravings Questionnaire (Cepeda-Benito *et al.*, 2000), or using visual analog scales (Nederkoorn *et al.*, 2000). The second category of outcome measures in studies on food cue reactivity entails peripheral physiological measures, such as insulin levels changes (Jastreboff *et al.*, 2013; Kroemer *et al.*, 2013), vagal responses (Udo *et al.*, 2014), heart-rate and heart-rate variability changes (Nederkoorn *et al.*, 2000), or salivation (Boyland *et al.*, 2017), among others. Finally, cue reactivity can be assessed with neurocognitive measures such as functional magnetic resonance imaging (fMRI) or electroencephalography (EEG) (van der Laan *et al.*, 2011), eye-tracking (Doolan *et al.*, 2014; Mehl *et al.*, 2017), or cognitive paradigms (e.g. Morys *et al.*, 2018; Oliva *et al.*, 2019), as reviewed below.

A number of fMRI studies have been designed around the hypothesis that individuals with obesity show neurobehavioral alterations in response to food. Perhaps the simplest way of testing this hypothesis is via the presentation of passively viewed food images. These designs allow the comparison between fMRI activity in response to visual food stimuli *vs* non-food items (usually objects). Such paradigms have been used to investigate potential behavioral causes of unhealthy weight gain, such as making sub-optimal food choices, low cognitive control in response to food stimuli or dysregulation in emotional control.

Investigations of cue reactivity leave a large freedom to the researchers in terms of designing experiments and analyzing resulting datasets (Smeets *et al.*, 2019). For example, while designing passive food picture viewing paradigms, one might consider contrasting reactivity to food pictures with non-food pictures (e.g. Davids *et al.*, 2010), or high-calorie with low-calorie food pictures (e.g. Frank *et al.*, 2014). Similarly, while investigating obesity-related changes in cue reactivity, experimenters tend to contrast obese groups with lean groups (e.g. Mehl *et al.*, 2018), correlate cue reactivity with obesity measures (e.g. BMI; Boswell and Kober, 2016) or only investigate cue reactivity in obese individuals without a control group (e.g. Luo *et al.*, 2013).

In the following sections of this article, we will focus on neurocognitive measures of cue reactivity in obesity, especially on neural correlates of passive food picture viewing. Specifically, we wish to test the hypothesis that there is either greater appetitive or reduced self-regulation response to food cues in obesity and that this is reflected in fMRI experiments. All the studies reviewed below meet *a priori* defined quality criteria. The most relevant criteria here are that all the studies examine fMRI differences between a condition of interest and a control condition and include obese and lean individuals.

## Literature review

### Passive image viewing in obesity

Studies investigating differences between obese and lean individuals in brain responses to food picture viewing have produced mixed findings. Bruce and colleagues showed that in obese children (n = 10), as opposed to healthy weight children (n = 10), food *vs* non-food pictures viewing elicits higher brain activations in the prefrontal cortex (PFC), both pre- and post-meal (Bruce *et al.*, 2010). Lower post-meal reductions in brain activity in obese *vs* lean children were also observed in the limbic and reward processing regions, e.g. the nucleus accumbens. The authors concluded that this shows hyperreactivity of obese children to food cues and reduced satiety effects. Such interpretation is in line with a study by Rapuano and colleagues (n = 78) showing that children with a higher genetic risk for obesity have increased activation of the nucleus accumbens to food advertisement (Rapuano *et al.*, 2017). Similarly, Davids and colleagues (n = 44) showed an increased activation in the dorsolateral prefrontal cortex (dlPFC) in response to food cues in obese *vs* lean children, but also increased caudate and hippocampal activations in lean *vs* obese children (Davids *et al.*, 2010). Studies in adults showed increased brain response to food pictures in obese *vs* lean individuals in the insula (Stoeckel *et al.*, 2008; Martin *et al.*, 2010; Oltmanns *et al.*, 2012; Scharmüller *et al.*, 2012), caudate (Rothemund *et al.*, 2007; Stoeckel *et al.*, 2008; Nummenmaa *et al.*, 2012), orbitofrontal cortex, amygdala, nucleus accumbens, anterior cingulate cortex, pallidum, putamen and hippocampus (Rothemund *et al.*, 2007; Stoeckel *et al.*, 2008; Martin *et al.*, 2010; Oltmanns *et al.*, 2012), or the PFC (Martin *et al.*, 2010; Dimitropoulos *et al.*, 2012). Generally, those regions are involved in processing of food cues (van der Laan *et al.*, 2011), but also play a role in dietary self-control (Han *et al.*, 2018; Neseliler *et al.*, 2019), reward and emotional processing, working memory (Pursey *et al.*, 2014), and interoception (Rahmani and Rahmani, 2019). Meta-analytical studies have shown that some of these brain regions, such as the nucleus accumbens, caudate, putamen or amygdala, are also activated in substance dependent individuals in response to drug cues (Tang *et al.*, 2012; García-García *et al.*, 2014), making a conceptual link between cue reactivity in addiction and obesity.

In contrast to these findings, decreased brain activation in obese *vs* lean individuals in response to food picture viewing has been shown in the anterior cingulate, lingual and superior occipital gyri (Heni *et al.*, 2014), superior frontal gyrus (Nummenmaa *et al.*, 2012), precentral gyrus, cingulate gyrus, dlPFC (Dimitropoulos *et al.*, 2012) and the temporal lobe (Martin *et al.*, 2010). Some of those regions overlap with those mentioned above and are also engaged in processing of food cues and dietary self-control (van der Laan *et al.*, 2011; Han *et al.*, 2018). Although reduced brain activation to food-cues may reflect impaired self-regulation, especially when it involves prefrontal areas, there remain inconsistencies in the neuroimaging findings. For instance, a number of studies did not show significant differences between lean and obese individuals for passive food picture viewing (Murdaugh *et al.*, 2012; García-García *et al.*, 2013b; Frank *et al.*, 2014; Doornweerd *et al.*, 2018; Morys *et al.*, 2018). In their systematic review, Pursey and colleagues outline a large number of brain structures that show higher brain activity in obese *vs* lean participants in response to visual food cues (Pursey *et al.*, 2014). This review, however, fails to differentiate between contrasts used in various studies (e.g. food > non-food cues, or high-calorie food > low-calorie food) and does not mention studies with no significant findings or findings where lean individuals had higher brain activity than obese individuals. A review by van der Akker and colleagues seems to further support the notion that obese individuals show higher responses to food cues than lean individuals (van den Akker *et al.*, 2014). A contrasting view is presented in a behavioral meta-analysis by Boswell and Kober, who found no effect of BMI group on cue reactivity measures (Boswell and Kober, 2016). We believe that such large inconsistencies in the literature warrant a well-controlled meta-analysis on cue responsivity differences in obese and lean individuals.

### Influence of food pictures on maladaptive behaviors in obesity

#### Reward sensitivity and decision-making.

Eating entails making food choices concerning what, how much, when or with whom to eat. These and other questions have been examined in the context of obesity. Specifically, studies have investigated whether obesity is associated with functional brain differences during food-related decision-making.

A study on children (n = 141), for instance, asked participants to perform food choices (either select the food displayed or reject it) under three conditions: the consideration of how healthy the food was, the consideration of tastiness, and a free (‘natural’) choice condition. Across all conditions, a higher BMI was associated with lower activity in the left dlPFC while selecting the food item displayed. This correlation was also found during the consideration of tastiness, which probably influenced the results most. The authors suggested that lower activity in the dlPFC might reflect lower cognitive control, which might jeopardize weight-loss interventions (van Meer *et al.*, 2019). These findings, however, might be difficult to integrate with findings from another study on portion size choices. In this study, participants (n = 36) were asked to select how much food they wanted to eat for lunch that day from a series of food items displayed in the fMRI. The right inferior frontal operculum showed higher fMRI activity in overweight participants relative to lean individuals (Veit *et al.*, 2019).

From a neuroeconomic perspective, food-related decision-making requires the integration of costs and benefits associated with each available choice (Essex and Zald, 2010). In this vein, auction tasks measure the willingness to pay for food and non-food items displayed during fMRI (Plassmann *et al.*, 2007). These tasks provide a direct measurement of current subjective value. An fMRI study using an auction task (n = 81) showed that obese and overweight individuals paid more money for highly palatable foods than lean participants. Moreover, participants with obesity showed higher fMRI activity in the caudate, accumbens and anterior cingulate cortex relative to lean participants (Verdejo-Román *et al.*, 2017). A similar auction task was used to show that these brain regions track the caloric density of food (Tang *et al.*, 2014). Together, these studies raise the possibility that individuals with obesity might attribute a greater subjective value to palatable food.

Finally, food cues can affect decision-making not only in food domains, but, among others, in monetary paradigms. In the field of obesity, one fMRI study has examined monetary delay-discounting in obese and lean individuals (Morys *et al.*, 2018, n = 36). Here, obese individuals showed less delay discounting (decreased impulsivity) when exposed to negative gustatory cues, which was related to decreased activity of the left dlPFC. This was, in turn, related to altered brain connectivity between the dlPFC and the ventromedial PFC, posterior cingulate gyrus and parietal cortex. Importantly, however, visual priming with food cues did not alter decision-making processes in obese or lean individuals. This suggests a lack of differences in visual food cue reactivity between obese and lean individuals, while pointing to the fact that more proximal cues (e.g. taste) might affect decision-making processes differently depending on weight status.

#### Inhibitory control paradigms.

In general, obese and lean individuals seem to differ in terms of their inhibitory control (Vainik *et al.*, 2013). A question remains, however, whether such differences are specific to food cues or can be generalized to other domains. Two of the tasks measuring inhibitory control are the Stop-Signal Reaction Task and Go/No-Go task. These tasks have been adapted from their original designs to incorporate food stimuli (Loeber *et al.*, 2012; Mühlberg *et al.*, 2016). In general, behavioral studies using the food versions of these tasks have reported similar performance in obese and lean participants (Loeber *et al.*, 2012; Mühlberg *et al.*, 2016). In a similar vein, a neuroimaging study from Carbine et al. compared fMRI responses to high *vs* low calorie foods using a Go/No-Go task (n = 54). The authors did not find an effect of obesity on this type of inhibitory control (Carbine *et al.*, 2018). Together, these findings suggest that obese and lean individuals have comparable inhibitory control responses towards food stimuli, both behaviorally and in terms of brain activity.

Another task, the approach/avoidance paradigm, allows testing food cue reactivity in the context of behavioral conflict (Mehl *et al.*, 2018, 2019). A behavioral study by Mehl and colleagues (n = 60) showed increased approach for food cues in obese compared to lean individuals (Mehl *et al.*, 2018). A follow-up fMRI study by the same group (n = 33) showed that approach bias for unhealthy food cues is related to increased activity in the right angular gyrus, while decreasing this bias by means of cognitive bias modification decreases the activity in the right angular gyrus and its connectivity to the right dorsal striatum (Mehl *et al.*, 2019). Those findings, however, were identified in a group of obese individuals only and not contrasted with a group of lean individuals.

### Influences on food cue reactivity independent of weight status

Factors beyond weight status might also influence food cue reactivity. Since the main focus of this paper is to investigate how weight status influences food cue reactivity, we will only briefly introduce other factors thought to influence neural responses to food cues. Lawrence and colleagues (n = 25) found that activity of the ventromedial PFC to food stimuli was positively related to self-reported hunger (Lawrence *et al.*, 2012). In the same study, activity of the nucleus accumbens predicted BMI in individuals with low self-control. Cosme and colleagues corroborated these results and found evidence that neural food cue reactivity was related to self-control (n = 94, Cosme *et al.*, 2019). Craving was also shown to be associated with neural food cue reactivity in the ventral striatum, anterior cingulate and orbitofrontal cortex (n = 60, Giuliani and Pfeifer, 2015). Interestingly, dietary restraint was associated with neural response to milkshake receipt in the orbitofrontal and dlPFC, but not to anticipated milkshake receipt or food pictures presentation (n = 39, Burger and Stice, 2011). Finally, and provided that emotions can affect eating behavior (Macht, 2008), the emotional context might also influence the neurobehavioral processing of food. In this vein, a study (n = 58) designed an emotional priming task to test whether the processing of food *vs* non-food pictures differed depending on the emotional context (i.e. negative, neutral or positive). The authors found that liking rates and amygdala activity differed according to emotional priming. Adiposity, however, measured using waist circumference, did not have an effect on the results (García-García *et al.*, 2019). In line with this, a study by Lopez and colleagues found that the activity of the inferior frontal gyrus in response to passive food viewing was lower in individuals with low desire to eat and high positive mood (n = 75, Lopez *et al.*, 2016).

### Meta-analysis aims

Conflicting findings in available literature regarding neural correlates of cue reactivity in obesity might arise from a number of factors: small sample sizes, control conditions used (e.g. low-calorie foods or non-food objects), lack of control conditions, lack of a control group, region-of-interest (ROI) *vs* whole-brain fMRI analysis and, others. To overcome these limitations and provide an objective assessment of cue reactivity in obesity, we perform a meta-analysis with a focus on studies investigating obese *vs* lean group differences in passive food picture viewing paradigms. Such analysis enables us to provide evidence for and elucidate mechanisms of food cue reactivity differences between obese and lean individuals. Based on previous reviews on the topic of obesity and reward processing (Stice *et al.*, 2009; García-García *et al.*, 2013a; van den Akker *et al.*, 2014; Pursey *et al.*, 2014), we hypothesized that obese individuals would show higher fMRI activity in response to visual food stimuli than lean individuals in predominantly reward-related brain regions, such as the nucleus accumbens, caudate, pallidum, putamen, ventromedial and orbitofrontal cortex. In contrast, we also hypothesized that lower brain activity in obese *vs* lean individuals will be observed in the dlPFC and the temporal cortex, perhaps reflecting reduced inhibitory control. These hypotheses are an extended version of our preregistered hypotheses.

## Materials and methods

Methods and analysis strategies used for the meta-analysis were preregistered prior to data collection. Protocols along with files used for the meta-analysis are available at https://osf.io/d53e6/.

### Study selection

Morys and García-García independently performed a literature search in the following scientific databases: PubMed, Scopus and Google Scholar. Keywords included 1) obesity-related terms, such as ‘obesity’, or ‘obese’ or ‘overweight’, 2) ‘food’ and 3) ‘fMRI’, or ‘MRI’, or ‘brain’. The results were then cross-validated between the authors and fitting articles were selected for further analysis. Each included study had to meet all of the following criteria: 1) studies using fMRI measured whole-brain activity as outcome measures 2) studies investigating group differences in cue reactivity between obese and lean individuals, 3) studies using food *vs* non-food pictures contrast, 4) studies reporting cluster peak coordinates and t-statistics or z-statistics for each cluster, if significant results were found. The meta-analysis also included articles reporting non-significant findings. We included articles that investigated cue reactivity in children and in adult samples. Main exclusion criteria were: 1) studies on clinical populations (e.g. individuals with depression or type II diabetes), 2) lack of a (control) group of lean individuals, 3) studies using fMRI paradigms other than passive viewing (these studies, however, have been reviewed in the section ‘Influence of food pictures on maladaptive behaviors in obesity’). To the best of our knowledge, all the studies included were performed on independent (i.e. non-overlapping) samples of participants.

### Seed-based d mapping meta-analysis

We conducted a meta-analysis using seed-based d-mapping (SDM, https://www.sdmproject.com/). This meta-analytic method allows the combination of fMRI studies using their cluster peak coordinates and effect sizes to find reliable, common patterns of activations in the brain for a specific effect of interest. It includes positive features from other meta-analytical methods, such as activation likelihood estimation or multi-kernel density analysis (e.g. weighting meta-analytic values by sample size of studies and using random-effects models), and extends those methods by adding certain improvements (Wager *et al.*, 2007; Radua and Mataix-Cols, 2009; Radua *et al.*, 2012). One of the improvements is the possibility of including both positive and negative effects in one analysis. Another one is including effect sizes from single studies to derive meta-analytic results.

Here, we investigated whether food cue reactivity differences between lean and obese individuals have consistent neural correlates. Additionally, because we recently found that some brain volume correlates of obesity might be age-dependent (García-García *et al.*, 2019), we investigated whether age influences brain mechanisms of cue reactivity in obesity. To this end, we performed meta-regression analysis with SDM software using age as a covariate of interest. For this analysis, a study by Martin and colleagues (Berridge *et al.*, 2010) was excluded because the authors did not report age of participants. We also investigated whether gender influenced weight group differences in neural responses to food *vs* non-food cues. Lastly, we performed a ROI analysis to investigate weight group differences in food cue reactivity in pre-defined ROIs derived from a previous meta-analysis investigating main effects of viewing food *vs* non-food stimuli (van der Laan *et al.*, 2011). This analysis deviated from our preregistration protocol and is reported as an exploratory analysis. The ROIs from this study include the orbitofrontal cortex, inferior frontal gyrus, insula, amygdala and several visual areas. In assessment of the results, we used threshold-free cluster enhancement (TFCE) multiple comparison correction with a 0.05 threshold (‘threshold-free’ in TFCE refers to the cluster-forming threshold; Smith and Nichols, 2009) after 1000 permutations with a cluster extent > 100 voxels. For significant clusters, we performed Egger test for asymmetry of the funnel plot to investigate potential publication bias.

## Results

### Articles included in the meta-analysis

In our database search, we identified 13 studies that fulfilled our criteria. Two of the studies investigated cue reactivity in children, while 11 studies investigated adults aged 18 to 75 years. The meta-analysis included 407 individuals. Details can be found in Table 1.

**Table 1.** Characteristics of studies included in the meta-analysis: we searched for fMRI studies examining whole-brain differences between obese and lean individuals during viewing of food pictures

Study	Year published	Sample size obese	Sample size lean	Mean age	BMI obese	BMI lean	Visual food stimuli	Control stimuli	Found significant clusters
Bruce *et al*.	2010	10	10	13	31.3	18.8	Appetizing low- and high-calorie food pictures	Pictures of animals	Yes
Davids *et al*.	2010	22	22	14	29.4	19.7	High-calorie food pictures	Neutral pictures of landscapes, buildings and work-related situations, pleasant pictures of babies, young animals, children playing.	Yes
Dimitropoulos *et al*.	2012	22	16	25	31.6	22.7	Low- and high-calorie food pictures	Furniture pictures	Yes
Doornweerd *et al*.	2018	16	16	50	28.4	24.4	Low- and high-calorie food pictures	Non-food items, such as trees, flowers, rocks, and bricks	No
Frank *et al*.	2014	11	11	40	40.2	21.4	Low- and high-calorie food pictures	Non-food pictures	No
Garcia-Garcia *et al*.	2013	18	19	33	34.9	22.4	Low- and high-calorie salty and sweet food pictures	Rewarding non-food stimuli	No
Heni *et al*.	2013	12	12	24	30.5	21.2	Low- and high-calorie food pictures	Pictures of objects with no association with eating	Yes
Martin *et al*.	2010	10	10	-	34.0	22.1	General food pictures	Gaussian blurred unrecognizable images of food and tools	Yes
Morys *et al*.	2018	24	27	27	34.3	22.1	Positive food pictures	Scrambled, unrecognizable versions of positive food pictures	No
Murdaugh *et al*.	2012	25	13	47	32.9	22.6	Low- and high-calorie food pictures	Pictures of cars	No
Nummenmaa *et al*.	2012	19	16	47	43.9	24.1	Appetizing and bland food pictures	Pictures of cars	Yes
Oltmanns *et al*.	2012	10	10	Age range: 20–45	35.1	-	High-calorie sweet and savory food pictures	Non-food objects of use	Yes
Rothemund *et al*.	2007	13	13	30	36.3	20.9	Low- and high-calorie food pictures	Neutral non-food related stimuli	Yes

### Meta-analysis results

**Fig. 1.**
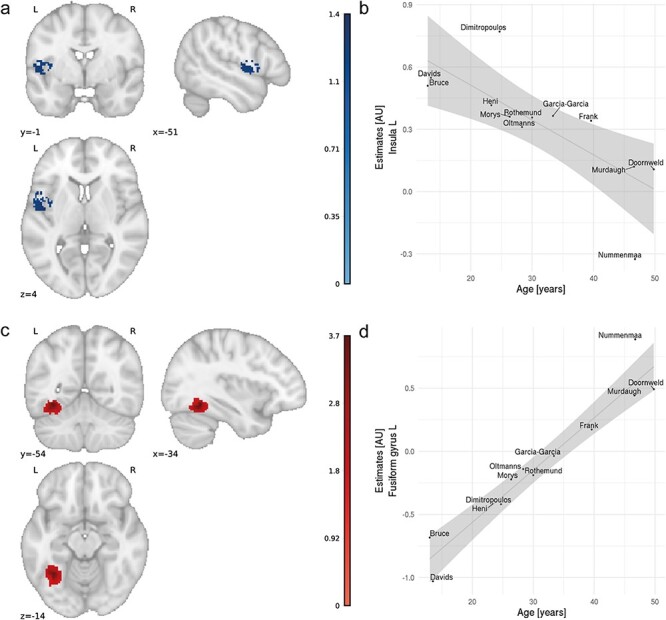
Results of meta-regression with age as a covariate of interest; a) cluster showing negative association with age for group differences between obese and lean groups in cue reactivity (z-scores); b) relationship between meta-analytic estimates from the cluster in the left insula and age; shaded area represents 95% confidence intervals; c) cluster showing positive association with age for group differences between obese and lean groups in cue reactivity (z-scores); d) relationship between meta-analytic estimates from the cluster in the left fusiform gyrus and age; shaded area represents 95% confidence intervals. AU, arbitrary units.

Contrary to our hypothesis, there were no significant differences in brain activity in obese *vs* lean participants for the contrasts of food > non-food pictures. However, meta-regression with age as a covariate revealed a significant cluster within the left posterior insula/Rolandic operculum, which was negatively related to age (peak MNI coordinates: −48, −8, 6; z-value: −1.416, size: 321 voxels; Figure 1, Table 2), and a cluster in the left fusiform gyrus which was positively related to age (peak MNI coordinates: −34, −54, −12; z-value: 3.681, size: 400 voxels; Figure 1, Table 2). Egger tests for funnel plot asymmetry performed for the peak voxel in those clusters did not show a significant publication bias (insula: t(10) = 0.02, *P* = 0.984; fusiform gyrus: t(10) = −0.02 *P* = 0.984). Upon further investigation, correlation analysis revealed that BMI in the lean groups was significantly related to mean age of samples included in the meta-analysis (r = 0.845, *P* = 0.001). This might mean that the clusters significantly related to age might in fact be related to the BMI of lean groups. We tested this in an additional meta-regression analysis where we included age as a predictor and regressed out effects of BMI of the lean group. In this analysis, we found that only the cluster in the left insula remained significantly related to age. This indicates that the cluster in the left fusiform gyrus was likely related to BMI of the lean group and not to the age of participants. We did not find an effect of gender on weight group food cue reactivity.

**Table 2.** Local peaks for clusters found in the meta-regression analysis

Peak voxel location	MNI coordinates			Z-value
	X	Y	Z	
*Fusiform gyrus L*				
Inferior longitudinal fasciculus	−34	−54	−12	3.681
Fusiform gyrus L	−34	−58	−14	3.547
*Insula L*				
Rolandic operculum L	−48	−8	6	−1.416
Superior temporal gyrus L	−50	4	0	−1.389
Superior temporal gyrus L	−48	0	0	−1.357
Insula L	−46	6	2	−1.336
Frontal aslant tract L	−48	4	6	−1.303
Inferior frontal gyrus, opercular part L	−54	8	6	−1.297
Inferior frontal gyrus, opercular part L	−50	10	8	−1.295
Rolandic operculum L	−56	2	6	−1.291
Fronto-insular tract L	−48	0	8	−1.286
Inferior frontal gyrus, opercular part L	−56	6	10	−1.278
Insula L	−38	0	4	−1.273
Rolandic operculum L	−44	−6	10	−1.269
Insula L	−44	−2	−2	−1.212

L, left; MNI, Montreal Neurological Institute.

In order to further evaluate the possibility of weight group effects on cue reactivity, we performed an exploratory analysis using ROIs derived from a previous food cue meta-analysis (van der Laan *et al.*, 2011). There were no significant differences in any ROIs, even with small-volume correction.

## Discussion

In this article, we first reviewed the existing literature and then performed a pre-registered meta-analysis on food cue reactivity in obese individuals. Previous theoretical work suggests that obesity is related to an enhanced salience response towards visual food stimuli (Berridge *et al.*, 2010). Moreover, greater brain responses to the sight of food have been proposed as a neural vulnerability factor for the development of obesity (Stice and Burger, 2019). However, although some exceptions exist (van Meer *et al.*, 2019), most studies examining fMRI reactivity to food cues during decision-making, reward sensitivity or cognitive control tasks found no effects of BMI (Carbine *et al.*, 2018; Morys *et al.*, 2018; Adise *et al.*, 2019). At the same time, results of neuroimaging studies investigating the influence of obesity on passive viewing of food cues are mixed. To investigate this issue further, we performed a meta-analysis of 13 studies with 407 participants investigating the influence of obesity on brain responses to food cues. We did not find evidence for overall altered cue processing in obese individuals. This is in line with a previous meta-analysis showing no influence of BMI on behavioral cue reactivity and craving (Boswell and Kober, 2016). However, we found two clusters in the brain that displayed an age-dependent relationship in this context: one in the left insula, which was negatively related to age, and one in the left fusiform gyrus, which was positively related to age. We did not find evidence for publication bias for either region. In our analyses, we only included studies that used appropriate control conditions (i.e. non-food cues) and control groups (lean individuals).

The first cluster was negatively related to age, which means that in children and young adults there was a weight group difference in left insula response to viewing food *vs* non-food pictures (Figure 1). This difference, however, decreased in older adults. Interestingly, a similar cluster in the left insula was previously observed to be activated for viewing food *vs* non-food pictures, independent of BMI (van der Laan *et al.*, 2011; Huerta *et al.*, 2014). Activation of the anterior and middle insula was also reported in studies investigating taste processing (Small, 2006), cephalic phase response (Tomasi *et al.*, 2009) or food craving (Pelchat *et al.*, 2004). Finally, insula was implicated in a meta-analysis of studies requiring subjects to intentionally regulate their level of craving in response to food (Han *et al.*, 2018). The effect was BMI dependent.

The second cluster in which we found age-dependent effects of cue reactivity in obese *vs* lean individuals was located in the left fusiform gyrus, a structure also related to food picture viewing (van der Laan *et al.*, 2011). The fusiform gyrus plays an important role in object recognition (Grill-Spector *et al.*, 2001) and in the case of food *vs* neutral stimuli viewing is possibly related to increased attention for and visual processing of food images (Killgore and Yurgelun-Todd, 2007; van der Laan *et al.*, 2011). Our age-dependent results are in line with a meta-analysis by van Meer and colleagues who showed that a similar brain region was activated to a higher degree in adults than in children/adolescents to food picture viewing (Van Meer *et al.*, 2014). In our study, weight group difference in activation of this brain region as a response to food cues increased with age (Figure 1). Van Meer explains this finding as meaning that food cues gain salience in adults as compared to children, which is reflected in higher activity in the left fusiform gyrus. In the context of our study, however, such an interpretation should be considered carefully, since a follow-up analysis revealed that the cluster in the left fusiform gyrus is likely related to BMI in the lean group and not to age *per se*.

Our findings stand in contrast with the conclusions from previous reviews on the topic of cue reactivity in obesity (Stice *et al.*, 2009; van den Akker *et al.*, 2014; Pursey *et al.*, 2014), which posit that there are indeed group differences in neural responses to food cues and that these differences present similarities with those found in substance addictions (García-García *et al.*, 2014). Interestingly, these reviews did not consider age effects and concluded that obesity is related to altered neural processing of food cues independent of age. This is contrary to our meta-analytic findings. These reviews, however, included studies using other stimuli than food pictures (e.g. gustatory stimuli, such as milkshakes), neglected findings from studies reporting no group differences and might have overinterpreted findings that are not methodologically robust. We believe that failing to include studies with null findings, coupled with reviewing studies that used inadequate statistical thresholding and underpowered sample sizes (Eklund *et al.*, 2016), might have led to incorrect conclusions. Such issues become evident when the neuroimaging literature is contrasted with the behavioral literature, as a meta-analysis performed by Boswell and Kober did not find evidence for the influence of BMI on food cue reactivity (Boswell and Kober, 2016). Here, the authors also showed that food cue reactivity predicts weight-gain. This is in line with other longitudinal findings in the neuroimaging domain showing that neural responses to food cues predict weight gain. For example, higher activity in the nucleus accumbens in response to food pictures tends to predict weight over a 6 months of follow-up (Demos *et al.*, 2012). In their study in 2018, Stice and Yokum showed that activity in the motor processing areas, but not in the striatum, predicts BMI gain over 3 years (Stice and Yokum, 2018). Conversely, fMRI has also been used in individuals undergoing weight-loss programs: food cue reactivity in brain areas related to reward (Murdaugh *et al.*, 2012), and self-regulation (Neseliler *et al.*, 2019) both predicted successful outcomes. Therefore, a question remains how cue reactivity can be independent of BMI and obesity status but also predict future weight fluctuations (Boswell and Kober, 2016). Van der Akker and colleagues claim that cue reactivity might lead to increased food intake and weight gain only in more impulsive individuals (van den Akker *et al.*, 2014). This suggests that there are multiple factors beyond BMI that need to be taken into account when investigating food cue reactivity. Some of those factors were reviewed in the Introduction to this article and include dietary restraint, self-control, hunger or food craving. Our meta-regression analysis shows that age might be another such factor. In addition, the nature of food cues (e.g. visual, olfactory or gustatory) might also differentially affect cue reactivity in obese individuals (Morys *et al.*, 2018). Another possibility is that pathological eating (such as binge eating or food addiction patterns) might mediate the effects of obesity on fMRI responses to the sight of food. Unfortunately, findings from fMRI studies on compulsive overeating are notably inconsistent (García-García *et al.*, 2020), and future research should consider both obesity and eating behavior to further test this possibility.

One limitation of the current meta-analysis is the use of BMI as a measurement of obesity. In some cases, individuals with high muscle mass who are not obese might be placed in the obese group based solely on BMI values. Further, only 3 studies included in the analysis reported the methods in which BMI was measured (self-reported *vs* measured), which might constitute an additional confound in the analysis as people tend to underreport their weight and overreport their height (Sherry *et al.*, 2007; Merrill and Richardson, 2009). However, without the explicit knowledge of the methods used to measure BMI and body composition of individuals included in the studies we were not able to correct for this in our analyses.

Overall, our review and meta-analysis show that there is scant evidence for food cue reactivity differences between lean and obese individuals. Our findings show that only two brain areas were related to weight group differences in visual processing of food cues and that these effects were age-related. Hence, additional factors contributing to neural correlates of food picture viewing in lean and obese individuals, such as age, self-control, food craving, impulsivity, hunger or dietary restraint need to be investigated in future studies. We propose that studies of better quality—using large sample sizes, appropriate statistical thresholding and ideally preregistered designs and analyses plans—should be performed to investigate in depth how those additional factors influence cue reactivity in obesity. In addition, a meta-analysis of behavioral cue-reactivity studies investigating effects of age might shed more light on and replicate the current neuroimaging-based findings.
